# Retention Strength after Compressive Cyclic Loading of Five Luting Agents Used in Implant-Supported Prostheses

**DOI:** 10.1155/2016/2107027

**Published:** 2016-10-16

**Authors:** Angel Alvarez-Arenal, Ignacio Gonzalez-Gonzalez, Hector deLlanos-Lanchares, Aritza Brizuela-Velasco, Javier Pinés-Hueso, Joseba Ellakuria-Echebarria

**Affiliations:** ^1^Department of Prosthodontics and Occlusion, School of Dentistry, University of Oviedo, Oviedo, Spain; ^2^Department of Oral Stomatology I, Faculty of Medicine and Dentistry, University of Basque Country, Bilbao, Spain; ^3^Private Practice, Bilbao, Spain

## Abstract

The purpose of this study was to evaluate and compare the retention strength of five cement types commonly used in implant-retained fixed partial dentures, before and after compressive cyclic loading. In five solid abutments screwed to 5 implant analogs, 50 metal Cr-Ni alloy copings were cemented with five luting agents: resin-modified glass ionomer (RmGI), resin composite (RC), glass ionomer (GI), resin urethane-based (RUB), and compomer cement (CC). Two tensile tests were conducted with a universal testing machine, one after the first luting of the copings and the other after 100,000 cycles of 100 N loading at 0.72 Hz. The one way ANOVA test was applied for the statistical analysis using the post hoc Tukey test when required. Before and after applying the compressive load, RmGI and RC cement types showed the greatest retention strength. After compressive loading, RUB cement showed the highest percentage loss of retention (64.45%). GI cement recorded the lowest retention strength (50.35 N) and the resin composite cement recorded the highest (352.02 N). The type of cement influences the retention loss. The clinician should give preference to lower retention strength cement (RUB, CC, and GI) if he envisages any complications and a high retention strength one (RmGI, RC) for a specific clinical situation.

## 1. Introduction

Currently, the high long-term survival of implants, especially in patients with good oral hygiene, makes the rehabilitation with a partial implant-supported fixed prosthesis an option frequently offered by the clinician and requested by the patient. There are only two systems to fix this kind of prosthesis to implants: screw-retained and cement-retained implant-supported restorations. Both systems have advantages and disadvantages and, depending on the particular clinical situation, one retention type could be more appropriate than the other [1]. The use of one or another retention system has long been discussed and still remains controversial among practitioners. There are not enough guidelines based on evidence-based clinical data to recommend a certain type of mechanism [2] and it seems to depend on the personal preferences and experience of the clinicians. Aesthetics, good mechanical environment, major passive fit, and occlusal control are the main advantages of a cement-retained implant-supported prosthesis, and the increased likelihood of peri-implantitis and the difficulty of retrievability are the main disadvantages. By contrast, the retrievability, ease of use, and greater stability in immediate loading and provisional restorations are the main advantages of a screw-retained implant-supported prosthesis and the mechanical-technical complications (screw loosening and others) are the main disadvantages [1]. However, recent systematic reviews [1–4] show similar survival rates, with no significant differences between the two retention types. Moreover, there is significantly lower total rate of technical and biological complications and more frequent fracturing of the veneering material in screw-retained implant-supported prostheses compared to the cemented ones [3].

When the clinician chooses a cement-retained implant-supported fixed partial prosthesis, he expects easy retrievability and enough retention strength to avoid decementation. However, there is a lack of sufficient scientific evidence relating to cement which fulfills these two conditions. Nowadays there is no consensus to establish which cement is the most appropriate for luting implant prostheses. Although some cement types are specifically manufactured to cement implant restorations, temporary or permanent cement types manufactured for the cementation of conventional dental-supported prostheses are widely used [5]. The dental literature evaluates and compares the retention of temporary cement types under different conditions and for different designs, and it has been reported that the most common complication is prosthesis debonding [6–12]. However, the high cost of implant-supported prostheses makes patients perceive this complication as unacceptable and obliges the prosthodontist to increase retention strength, using permanent cement or a screw-retained prosthesis [6, 13–16]. Regardless of the geometry of the abutment and other mechanical and biological factors that can strongly influence the retentiveness of the cement, the retention strength and degree of retrievability of the cemented restorations depend on the chosen cement, and, by definition, permanent cement types are those that offer the greatest retention. However, the greatest disadvantage of permanent cement is the lack of retrievability, and yet their clinical use is common, with frequent research carried out to compare their retention strength [6, 10, 11, 13, 17–23]. Urethane-based resin, compomer, glass ionomer, resin-modified glass ionomer, and resin composite are some of the kinds of cement available and used clinically to cement fixed partial prostheses to implant abutments [5].

A large number of literature references compare the retentiveness of these cement types in implant dentistry with different types of prosthetic restorations and abutments, different conditions of compressive loading, and different simulating intraoral conditions [6, 9–11, 17–29]. By contrast, there are very few references comparing retentiveness before and after a cyclic compressive loading which simulates mastication to decide whether the final retention of these cement types after a long period of mastication is enough to support the retrievability and at the same time keep the restoration in place [8, 18, 20, 26]. Knowledge of these data may be useful to clinicians to decide which cement to use if they need to retrieve the restoration. The aim of this study was to assess and compare retention before and after a cyclic compressive loading of five commonly used dental cement types when cementing cast crowns to implant abutments in relation to the retrievability of the restoration. The null hypotheses were that retention loss after the cyclic compressive loading of the cement types with higher tensile bond strength (resin composite and resin-modified glass ionomer) would be similar to cement types with lower tensile bond strength, such as glass ionomer, compomer, and urethane-based resin, and five cement types would allow the retrievability of implant-supported cemented prostheses.

## 2. Materials and Methods

### 2.1. Preparation of Specimen

**Figure 1:**
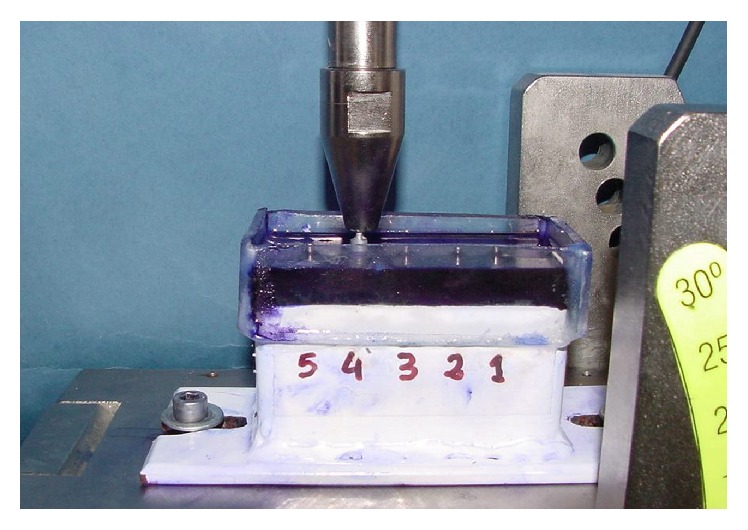
Working model. Cyclic compressive load test.

The working model was a rectangular metal frame filled with self-curing acrylic rose with a transparent methyl-methacrylate recipient on top. In the resin block, 5 holes (10 to 12 mm apart) were made and, with the aid of a parallelometer, 5 implant analogs 4 × 10 (Stark-D; Sweden & Martina, Due Carrare, Italy) were placed in them. A titanium solid abutment, tapered 6 degrees and 7 mm in height, was screwed to each implant using a manual torque controller at 30 Ncm and the abutments were numbered from 1 to 5 on the outside of the model (Figure 1). 50 individual premachined castable copings were cast in nickel-chromium alloy (Wiron 99; Bego, Lincoln, RI). On its occlusal surface, each coping was provided with a loop for attachment to the tensile testing machine. Copings were numbered 1 to 50 and were randomized into the five cement types to be assessed (*n* = 10); moreover, each coping from each cement group was randomly assigned to each abutment.

### 2.2. Luting of Copings

Five kinds of cement were evaluated: resin-modified glass ionomer cement (Fujicem Automix; GC Europe, Leuven, Belgium); resin composite cement (Multilink Implant; Ivoclar, Schaan, Liechtenstein); glass ionomer cement (Fuji I Capsule; GC Corporation, Tokyo, Japan); compomer cement (Stay Bond KDM; Kalma, Madrid, Spain); and urethane-based resin cement (Premier Implant Cement; Premier, Plymouth Meeting, PA) (Table 1). All the copings were cemented by the same operator, who, like the data analyst, was unaware of the cement being used (double blind). The cementation protocol for the five types of cement was in accordance with the manufacturers' instructions; also, mixing error and mixing time were minimized by using Automix syringes or mechanical mixing capsules. The copings were completely covered with the luting agent and were placed on the abutment by applying firm finger pressure for 20 seconds, followed by a 5 kg compressive axial load for 10 minutes. Excess cement was removed from the margin at 30 minutes cementation and the complete seating of the coping on the complex implant-abutment was visually checked for marginal integrity and to ensure there was no marginal gap and after another 30 minutes the initial tensile test was carried out.

### 2.3. Testing of Tensile Strength

**Figure 2:**
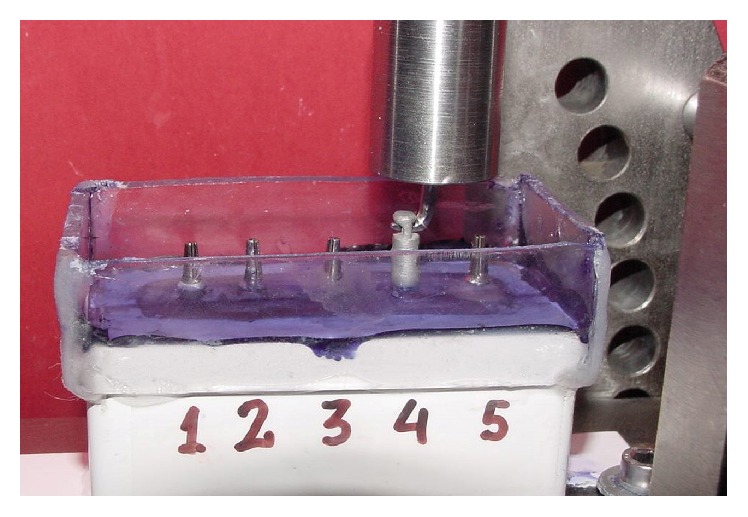
Tensile test after cyclic compressive loading.

**Figure 3:**
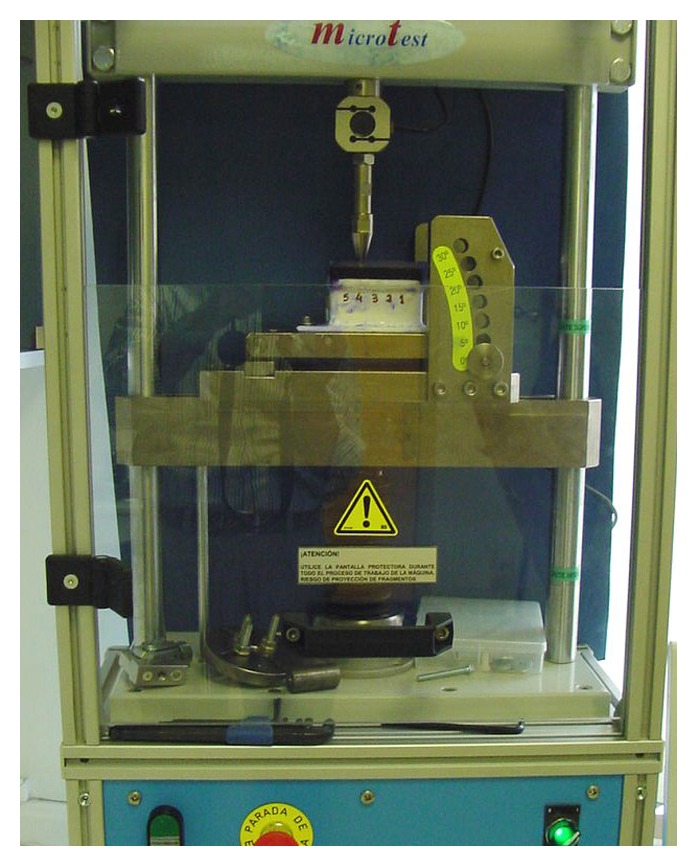
Universal tensile-compressive test machine.

After the initial tensile test, copings and abutment-implants were cleaned with distilled water for 10 minutes, using a Hollenback carver and an ultrasonic bath. Complete removal of the cement was confirmed with a 4x magnifying glass. The copings were then cemented again using the procedure described above. The container of the methyl-methacrylate working model was then filled with a saturated physiological saline solution colored with crystal violet to cover two-thirds of the height of the copings and the compression test was carried out. Each coping from each cement group was subjected to a cyclic compressive load of 100 N, at a frequency of 0.72 Hz, until 100,000 cycles were completed, to simulate 2-3 months of average human masticatory function (Figure 1). After the compression test, the liquid medium was removed with a syringe and then the tensile test was performed (Figure 2). A model EM1/5FR universal testing machine (Microtest, Madrid, Spain) and SCM3000 software (Microtest, Madrid, Spain) were used to apply the compressive and tensile forces to the copings (Figure 3).

### 2.4. Statistical Analysis

In order to examine the equality of mean retention values between groups before and after compressive loading in the cement types, one way ANOVA tests were performed. Afterwards the post hoc Tukey test was used to find which cement types mean retention values were significantly different from each other. All the statistical tests were conducted at *p* < 0.05 significance level.

## 3. Results

Table 2 shows retention strength values before and after cyclic compressive loading and the retention index of the five cement types tested. Before the application of the compressive load, resin-modified glass ionomer and resin composite cement showed the greatest retention strength. The values of resin composite cement were statistically significantly different from the other four cement types of the study. The temporary urethane-based resin cement had the next highest retention value. The retention of this cement was approximately half that of the resin composite and resin-modified glass ionomer cement types, slightly higher than compomer cement retention and 75% greater compared to the retention of glass ionomer cement, with no statistically significant differences being revealed by the Tukey post hoc test.

After the compressive test, the retention of all cement types was lower compared to the initial retention, with statistically significant differences for urethane-based resin, compomer, and glass ionomer cement types. The retention index measures the percentage rate of change of retention before and after compressive loading and is calculated using the following formula: Retention  Index = (1 − retention  after  load/retention  before  load) × 100.

According to this index, the Premier Implant Cement, Stay Bond, and Fuji I Capsule cement types show the greatest percentage loss of retention strength compared to Fujicem Automix and Multilink Implant cement types. Also, after compressive loading, the Multilink Implant shows the greatest retention strength with statistically significant differences from the other four cement types, as does the Fujicem Automix cement from the Fuji I Capsule and Premier Implant Cement.

## 4. Discussion

### 4.1. Clinical and Biologic Implications

This study was conducted to determine the loss of retention strength in five cement types commonly used in clinical practice after cyclic compressive loading equivalent to two to three months of mastication. The results obtained prevent us from accepting the stated null hypothesis, since, after compressive loading, cement types of higher tensile strength showed significant differences in retention strength compared to lower tensile strength cement types, which, moreover, do not permit retrievability of the cement-retained implant-supported prosthesis.

Before application of a compressive load, five cement types showed enough retention strength to keep the crowns in place, a force of between 100.41 N (Fuji I Capsule cement) and 443.15 N (Multilink Implant cement) being required to dislodge them. This great retentiveness of the resin composite cement agrees with the high retention strength values of the resin composite cement types, with or without 10-MDP monomer (10-methacryloyloxydecyl dihydrogen phosphate), quoted in other studies [10, 11, 17, 21–23]. Likewise, the resin-modified glass ionomer cement (Fujicem Automix) showed retention strength values similar to those found by other authors but also different from other studies that report higher or lower values [10, 11, 17, 19, 20, 22, 23]. In the same way, the Premier Implant Cement and the Fuji I Capsule cement types revealed initial retentiveness values close to those cited in the dental literature or, conversely, higher or lower [9, 15, 17, 19, 21–24, 27–29]. Although retention strength data have not been found for compomer cement in the literature, its initial mean retention strength values, before loading, are in the range reported by other authors for glass ionomer cement [10, 19, 22, 23]. Regardless of the nature of the cement types, the use of different alloys/materials, tapers, heights, diameters, roughness and surface treatment abutments, mixes and luting, environment, and waiting times for the tensile test differences, as well as commercial differences in cement composition, may explain the differences in the initial retention strength values of these dental cement types reported in the literature [9, 11, 15, 16, 21, 22, 26].

Furthermore, independently of factors that may influence the retention strength before compressive loading, the results obtained showed that any cement of this study has enough initial strength to lute a single implant or conventional prosthesis. However, the high retention values recorded with the Multilink Implant and Fujicem Automix cement showed that these cement types are more suitable for cementing unfavorable fixed prostheses (low height or excessively tapered abutments), while the Premier Implant Cement, Fuji I Capsule, and compomer cement types would be more appropriate in cases with better prosthetic retention characteristics.

On the other hand, if the retentive strength is an indirect measure of the ability of the prosthetic restoration to withstand dislodgement forces, in this study this ability has decreased markedly for the five cement types after compressive loading corresponding to 2-3 months of simulated chewing, with differences in the percentage of retention loss. However, the Multilink Implant and Fujicem Automix cement showed a high retention strength that does not favor the retrievability of the restoration; 174.50 N would be required to dislodge the crowns cemented with resin-modified glass ionomer (Fujicem Automix) and double that strength for the ones cemented with resin composite (Multilink Implant). Therefore, these cement types should not be used in those cases where the dentist needs to retrieve the crowns, unless the technique of cement-screw-retained restoration is used, which consists of making a hole in the occlusal surface of the crown to allow direct access to the abutment screw. This technique does not appear to significantly decrease the cement retention strength [13]. For the compomer (Stay Bond KDM) and glass ionomer (Fuji I Capsule) cement types, the retention strength decreased after compressive loading by about half (50%) and by three-fifths (64%) in the case of resin urethane-based (Premier Implant Cement) cement. Despite this higher percentage of retention loss, resin urethane-based cement maintained a retention strength similar to that of compomer cement, and both had retention values 50% higher than the glass ionomer cement. These results confirmed that the mechanical stress of the cyclic compressive loading reduces the retentiveness of the cement types, though not to the same degree.

Also, these three cement types showed retention strength values after compressive cyclic loading of between 50.35 N (Fuji I Capsule cement) and 75.12 N (Stay Bond cement), which can ensure retrievability but may not prevent dislodgement of the crown during function.

When the patient's treatment is a cemented implant-supported fixed partial denture, one of the main concerns of the clinician, in addition to preventing peri-implantitis and bone loss around implants, is to use cement that allows restoration retrievability in the event of a complication arising. The data of this study showed that despite the large percentage of retention loss of the five cement types, only the use of the Premier Implant Cement, Fuji I Capsule, and Stay Bond KDM cement types would help prosthesis retrievability, as a maximum force of just 75.12 N would be sufficient to dislodge the crowns. However, it is not possible to assert whether the retention strength of these three cement types is enough to keep the restoration in place. In the dental literature no data were found regarding the minimum retention strength values of cement needed to keep a crown in place. However, knowing the results of this study can be useful for practitioners if they want cement that allows them to retrieve the restoration. In contrast, if the retrievability is not a problem and the clinicians wish for cement to keep the restorations in place for a long time without frequent debonding, the Fujicem Automix and Multilink Implant cement types are suitable. This is confirmed by the 174.50 N and 352.02 N needed, respectively, to debond the crowns with these two cement types.

Therefore, in accordance with the results of this research, when the dentist suspects a short-term biological or mechanical-technical complication of any kind, he must choose the urethane-based resin, compomer, or the glass ionomer cement types. If, on the other hand, the dentist does not envisage any complication or the abutments have poor retention forms, resin composite (Multilink Implant) or resin-modified glass ionomer (Fujicem Automix) cement types are the most suitable luting agents. Although neither of these two cement types favors retrievability, the clinician should know that choosing the resin-modified glass ionomer cement allows a greater possibility of retrievability than does the resin composite cement.

### 4.2. Limitations and Justification of the Experimental Design

This study is an in vitro test that simulates the influence of mastication on dental cement retention strength. During mastication, complex strength patterns occur in different directions with a combination of compression, tensile, and shear stress, all contributing to crown debonding. This situation is not easily reproducible in vitro and constitutes a limitation, since only axial compressive loading and a pure tensile test were used. Furthermore, inter- and intraindividual mastication variability associated with the type of food, age, sex, missing teeth, and use of prosthesis or joint and muscle pathology means that there is great variability in the number of cycles corresponding to the average daily, weekly, or annual human masticatory function. In this trial, the number of compressive cycles coincides with one study and was lower than in other studies [8, 20]. A load of 100 N was applied, a value close to that produced in the front part of the mouth and not far from the 75–110 N of other studies [8, 26]. This trial did not take into account the temperature, the tests having been performed at room temperature of between 22 and 24 degrees. All of this constitutes a limitation, because although not all cement types are affected to the same extent by temperature changes, it has been noted that thermal cycles decrease the retention strength of cement types, when there are large differences between thermal expansion coefficients of the metal casting and cement [20, 27]. Furthermore, the lower values of the retention strength of the glass ionomer (Fuji I Capsule) cement both before and after the compressive cyclic loading may be because of the little time (one hour) that has elapsed between the cementing of the copings and the beginning of the initial tensile and compressive load tests as well as the humid environment of the experiment. It is well known that glass ionomer and resin-modified glass ionomer cement types have, in addition to the ability to absorb water, a very peculiar setting process, that is, a prolonged acid-base reaction taking days or weeks to reach its maximum strength [30, 31]. This is also a relative limitation, as the patients treated with a conventional or unconventional fixed prosthesis cemented with this kind of cement begin to chew 1–3 h after cementation.

The reuse of the copings for the tensile test after compressive loading and of the abutments for each type of cement can be a limitation, because although there is insufficient information available on the effect of this reuse on retention strength, there is one study that indicates that this strength may be altered due to luting and removing cement from abutments and metal castings [11]. Future research should take these limitations into account by increasing the sample size and standardizing the experimental design with in vitro conditions as close as possible to the oral environment during mastication. Furthermore, in vitro studies are unable to replicate exactly all of the oral environment variables during mastication, such as temperature changes, salivary characteristics (pH, buffering capacity, and flow rate), characteristics of food, occlusal forces, and tongue movements, all of which may influence dental cement retention strength. Therefore, no clear correlation can be established between the results obtained and clinical practice performance.

## 5. Conclusions

Within the limitations and the in vitro conditions used in this study and in accordance with the results obtained, the following conclusions may be drawn:

(1) The cyclic compressive load decreases the retention strength of the five cement types. The resin composite (Multilink Implant) and resin-modified glass ionomer (Fujicem Automix) cement types recorded the lowest percentage of retention loss and the resin urethane-based (Premier Implant Cement) cement the highest. (2) The glass ionomer, compomer, and urethane-based resin cement types may favor the retrievability of implant-supported cemented prostheses. In contrast, the resin composite and the resin-modified glass ionomer keep the crowns in place, preventing their dislodgement during function. (3) Since the type of cement influences the retention loss, clinicians should give preference to a lower retention strength cement if complications are envisaged and a high retention strength cement for a specific clinical situation.

## Figures and Tables

**Table 1:** Features and composition of the dental luting agents used in this study.

Cement type	Brand	Manufacturer	Composition
Resin-modified glass ionomer cement	Fujicem Automix	GC Europe Leuven, Belgium	Fluoroaluminosilicate glass, aqueous solution of polycarboxylic acid modified with methacrylate groups, HEMA
Resin composite cement	Multilink Implant	Ivoclar Vivadent Schaan, Liechtenstein	Matrix: dimethacrylate resin, HEMA Fill: barium glass, ytterbium fluoride
Glass ionomer cement	Fuji I Capsule	GC Corporation Tokyo, Japan	Powder: silica glass, alumina glass Liquid: polyacrylic acid, tricarboxylic copolymer acid
Compomer cement	Stay Bond	KDM, Kalma Madrid, Spain	UDMA, bifunctional monomers Fill: silica and alumina glass
Resin urethane-based cement	Premier Implant Cement	Premier Plymouth Meeting, PA, USA	Base: UDMA, TEGDMA, HEMA Catalyst: UDMA, TEGDMA, benzoyl peroxide

HEMA: hydroxyethylmethacrylate. UDMA: urethane dimethacrylate. TEGDMA: triethylenglycoldimethacrylate.

**Table 2:** Mean tensile retention strength of tested cement types before and after cyclic compressive loading.

Cement type and brand	Retention before loading (N) (SD)	Retention after loading (N) (SD)	*p* value	Retention index (%)
Resin-modified glass ionomer (Fujicem Automix)	253.35^*∗*^ (85.38)	174.50^*∗∗∗*^ (92.09)	0.155	31.12
Resin composite (Multilink Implant)	443.15^*∗∗*^ (69.41)	352.02^*∗∗*^ (76.05)	0.055	20.56
Glass ionomer (Fuji I Capsule)	100.41 (33.55)	50.35 (30.37)	0.007	49.86
Compomer (Stay Bond)	161.13 (53.34)	75.12 (72.63)	0.005	53.38
Resin urethane-based (Premier Implant Cement)	174.76 (45.59)	71.25 (73.86)	0.005	59.23

Values of retention in Newton; *n* = 10. Standard deviation in brackets. The Tukey test: ^*∗*^statistically significant differences with respect to glass ionomer cement; ^*∗∗*^statistically significant differences with respect to the other four cement types; ^*∗∗∗*^statistically significant differences with respect to glass ionomer and resin urethane-based cement types.
